# Conversion of CH_4_ and Hydrogen Storage via Reactions with MgH_2_-12Ni

**DOI:** 10.3390/mi14091777

**Published:** 2023-09-16

**Authors:** Young Jun Kwak, Myoung Youp Song, Ki-Tae Lee

**Affiliations:** 1 Division of Advanced Materials Engineering, Jeonbuk National University, 567 Baekje-daero, Deokjin-gu, Jeonju 54896, Republic of Korea; twistking18@nate.com (Y.J.K.); ktlee71@jbnu.ac.kr (K.-T.L.); 2 Hydrogen & Fuel Cell Research Center, Engineering Research Institute, Jeonbuk National University, 567 Baekje-daero, Deokjin-gu, Jeonju 54896, Republic of Korea; 3 Department of Energy Storage/Conversion Engineering of Graduate School (BK21 FOUR), Jeonbuk National University, 567 Baekje-daero, Deokjin-gu, Jeonju 54896, Republic of Korea

**Keywords:** conversion, CH_4_, hydrogen production, adsorption, hydrogen storage, MgH_2_-12Ni

## Abstract

The main key to the future transition to a hydrogen economy society is the development of hydrogen production and storage methods. Hydrogen energy is the energy produced via the reaction of hydrogen with oxygen, producing only water as a by-product. Hydrogen energy is considered one of the potential substitutes to overcome the growing global energy demand and global warming. A new study on CH_4_ conversion into hydrogen and hydrogen storage was performed using a magnesium-based alloy. MgH_2_-12Ni (with the composition of 88 wt% MgH_2_ + 12 wt% Ni) was prepared in a planetary ball mill by milling in a hydrogen atmosphere (reaction-involved milling). X-ray diffraction (XRD) analysis was performed on samples after reaction-involved milling and after reactions with CH_4_. The variation of adsorbed or desorbed gas over time was measured using a Sieverts’-type high-pressure apparatus. The microstructure of the powders was observed using a scanning transmission microscope (STEM) with energy-dispersive X-ray spectroscopy (EDS). The synthesized samples were also characterized using Fourier transform infrared (FT-IR) spectroscopy. The XRD pattern of MgH_2_-12Ni after the reaction with CH_4_ (12 bar pressure) at 773 K and decomposition under 1.0 bar at 773 K exhibited MgH_2_ and Mg_2_NiH_4_ phases. This shows that CH_4_ conversion took place, the hydrogen produced after CH_4_ conversion was then adsorbed onto the particles, and hydrides were formed during cooling to room temperature. Ni and Mg_2_Ni formed during heating to 773 K are believed to cause catalytic effects in CH_4_ conversion. The remaining CH_4_ after conversion is pumped out at room temperature.

## 1. Introduction

The global economy is developing gradually, and consequently, the global energy demand is constantly growing. Energy is supplied from fossil fuels such as coal, crude oil and natural gas, which are finite on Earth. The use of fossil fuels as an energy source has led to global warming and climate change. To solve these problems, alternative energy sources should be developed.

As alternative energy sources, we can consider solar energy, wind energy, geothermal energy, hydropower, ocean energy and bioenergy.

Many researchers are interested in the production and storage of hydrogen based on the use of alternative renewable energy sources. In a renewable energy-based hydrogen economy, the distribution of hydrogen from the producer to consumer is currently a key missing technology.

Hydrogen energy is the energy produced via the reaction of hydrogen with oxygen. The reaction of hydrogen with oxygen simultaneously produces water. Hydrogen energy is considered one of the potential substitutes to overcome the growing global energy demand [1,2]. Hydrogen energy is believed to lead to a ‘hydrogen energy economy’ society.

Electrochemical devices, particularly fuel cell systems, have great potential to revolutionize the way power is produced and utilized. Direct electrochemical production promises greater energy efficiency, less dependence on non-renewable resources and less environmental impact. However, fundamental challenges remain in developing the material systems necessary to achieve the required levels of performance and durability and make solid oxide fuel cell technology a reality.

Fuel cells are energy conversion devices that produce electricity by electrochemically combining fuel and oxidizing gases across an electrolyte [3]. The scientist William Grove first demonstrated the fuel cell concept and associated electrochemical processes in 1839 [4]. He reversed the electrolysis process—where hydrogen and oxygen recombine—and showed that a small electric current could be produced [5]. Although the concept was demonstrated more than 180 years ago, fuel cells have only recently attracted serious interest as an economically and technically applicable power source.

As a new generation of power sources compared with conventional energy systems, fuel cells have a number of advantages, thanks to which they have gained widespread recognition. A key feature of a fuel cell system is its high energy conversion efficiency. Since the fuel cell converts the chemical energy of the fuel directly into electrical energy, its conversion efficiency is not subject to the Carnot limitation [5]. Other advantages over conventional power production methods include modular construction, high efficiency at partial load, minimal location constraints, cogeneration potential and much lower production of pollutants [5].

Hydrogen is usually stored in a gaseous state under high pressure and in a cryogenic liquid state [6]. Storing gaseous hydrogen has disadvantages such as safety issues, high cost and hydrogen’s embrittlement of storage tank materials. Storage of hydrogen in a cryogenic liquid state has drawbacks such as thermal losses in the case of an open system, safety and cost of liquefaction.

Solid-state hydrogen storage using materials such as metal hydrides has advantages such as high gravimetric and volumetric storage capacities and safety, as metal hydrides can absorb and release hydrogen at relatively low pressures. Hydrogen is bound by chemical or physical forces in hydrogen storage based on solid-state materials. The technique of storing hydrogen in a solid state has become very attractive [7] and is the subject of studies by many researchers [8,9,10,11,12].

The hydrogen-storage capacity of magnesium is high, its price is low and its reserves in the Earth’s crust are large. However, its reaction rate with hydrogen is low even at a relatively high temperature such as at 573 K [13]. A lot of work on improving the hydriding and dehydriding rates of magnesium has been put into alloying magnesium with certain metals [14], such as Cu [8], Ni [9,10], Ti [11], Sn [15], V [16] and Ni and Y [17].

Reilly et al. [9] and Akiba et al. [10] improved the reaction kinetics of Mg with H_2_ by preparing Mg-Ni alloys. Song et al. [18] increased the hydriding and dehydriding rates of Mg via the mechanical alloying of Mg with Ni under an Ar atmosphere. Bobet et al. [12] improved the hydrogen-storage properties of both magnesium and Mg + 10 wt% Co, Ni, and Fe mixtures by means of mechanical milling under H_2_ (reaction-involved milling) for a short time (2 h). In our previous work [19], samples with the compositions of 94 wt% MgH_2_ + 6 wt% Ni, 88 wt% MgH_2_ + 12 wt% Ni, 85 wt% MgH_2_ + 15 wt% Ni and 82 wt% MgH_2_ + 18 wt% Ni were prepared by means of reactive mechanical grinding. Then, the variations of the hydriding and dehydriding properties in the first hydriding-dehydriding cycle with Ni content were investigated. The sample with the composition of 88 wt% MgH_2_ + 12 wt% Ni had the highest hydriding rate and the largest quantity of hydrogen absorbed for 60 min. Therefore, we selected this sample (named MgH_2_-12Ni) as the suitable alloy.

There are three types of methane reforming: steam reforming, autothermal reforming and partial oxidation. These are chemical processes that can produce pure hydrogen gas from methane using a catalyst. Most methods rely on exposing methane to a catalyst (usually nickel) at high temperatures and pressures [20].

Milling particles in a hydrogen atmosphere (reaction-involved milling) generates defects, causes cracks and creates clean surfaces, and reduces particle size. In this way, reaction-involved milling puts the sample in a state that is readily operable with gas; defects can act as active nucleation sites, clean surfaces show high reactivity with gas, and particle size reduction shortens the diffusion distances of atoms.

The main obstacles that need to be overcome in the future in order to move to the hydrogen economy society are the development of hydrogen generation and storage methods. In this work, a new study on CH_4_’s conversion to hydrogen and the storage of hydrogen was performed using a magnesium-based alloy. MgH_2_-12Ni (with the composition of 88 wt% MgH_2_ + 12 wt% Ni) was prepared in a planetary ball mill by means of reaction-involved milling. X-ray diffraction (XRD) analysis was performed on samples after reaction-involved milling and after reactions with CH_4_. The variation of adsorbed or desorbed gas over time was measured using a Sieverts’-type high-pressure apparatus under a methane pressure of 12 bar at 773 K. The microstructure of the powders was observed using a scanning transmission microscope (STEM) with energy-dispersive X-ray spectroscopy (EDS). The reacted samples were also characterized using Fourier transform infrared (FT-IR) spectroscopy.

One of the studies aimed at the practical application of fuel cells is the production and storage of hydrogen. We were able to generate hydrogen from CH_4_ and at the same time store it as a nano-sized metal hydride. The results of this work can be applied to the production and storage of hydrogen, which can be used for supplying hydrogen to fuel cells. The materials developed in our work are believed to be used for motive power fuel and portable appliances as mobile applications, transport and distribution as semi-mobile applications, and industrial off-peak power H_2_-generation, hydrogen-purifying systems and heat pumps as stationary applications.

## 2. Materials and Methods

MgH_2_ (magnesium hydride, 98%, Alfa Aesar, Ward Hill, MA, USA), Ni (average particle size 2.2–3.0 µm, purity 99.9% metal basis, C typically < 0.1%, Alfa Aesar, Ward Hill, MA, USA) and CH_4_ (purity 99.95%, O_2_ < 5 ppm, N_2_ < 100 ppm, H_2_O < 5 ppm, H_2_ < 1 ppm, THC (total hydrocarbon content) < 5 ppm, and CO/CO_2_ < 1 ppm, Korea Noble Gas Co. Ltd., Daejeon, South Korea) were used as starting materials.

A mixture with the composition of 88 wt% MgH_2_ + 12 wt% Ni (total weight of 8 g) was placed in a hermetically sealed stainless-steel container with 105 hardened steel balls (total weight of 360 g). The sample-to-ball-weight ratio was 1/45. The samples were handled in a glove box under Ar to prevent oxidation. MgH_2_-12Ni with the composition of 88 wt% MgH_2_ + 12 wt% Ni was prepared in a planetary ball mill (Planetary Mono Mill; Pulverisette 6, Fritsch, Weimar, Germany) by milling at a disc revolution speed of 400 rpm under a high-purity hydrogen gas of 12 bar for 6 h. Pure MgH_2_ was also milled under the same conditions and named as milled MgH_2_.

The variation in the amount of adsorbed or desorbed gas over time was measured by means of the volumetric method in a Sieverts’-type high-pressure apparatus described previously [21]. This apparatus is composed of three parts: a reactor containing the sample, a gas-supplying part and a part of a standard volume with a known volume used to measure the amount of adsorbed or released gases. The amount of adsorbed gas was measured based on changes in the pressure of the standard volume over time. The standard volume pressure decreases as some gas is transferred to the reactor to compensate for the gas pressure drop in the reactor due to gas adsorption. The amount of desorbed gas was measured based on changes in the pressure of the standard volume over time. The pressure of the standard volume increases as some gas is transferred from the reactor to the standard volume to remove the amount of gas from the reactor (whose pressure increases due to gas desorption). The amount of sample (MgH_2_-12Ni) used for these measurements was 0.5 g.

For the reaction of methane in milled MgH_2_ and MgH_2_-12Ni, we chose a temperature of 773 K, which is not too high compared with the temperature of metal hydride formation. This temperature is lower than the temperature at which CH_4_ conversion was performed in the reported works [20]. We chose a gas pressure of 12 bar at 773 K because too high a gas pressure causes leakage in the parts of the Sieverts’-type high-pressure apparatus.

X-ray diffraction (XRD) patterns of samples after reaction-involved milling and after adsorption–desorption were obtained in a powder diffractometer Rigaku D/MAX 250 (Tokyo, Japan) with Cu Kα radiation. XRD pattern analysis was performed using the MDI JADE 5.0 program. Data from the JCPDS PDF-2 2004 card of the International Centre for Diffraction Data (ICDD) were used to identify the phases. Reacted samples were also characterized using Fourier transform infrared (FT-IR) spectroscopy (Frontier, PerkinElmer, Shelton, CT, USA). Powder microstructures were observed using a high-resolution transmission electron microscope (HR-TEM) with energy-dispersive X-ray spectroscopy (EDS) (Titan G2 Cube 60-300, FEI company (Field Electron and Ion Company, FEI, Hillsboro, OR, USA)) operated at 80 kV.

## 3. Results and Discussion

**Figure 1:**
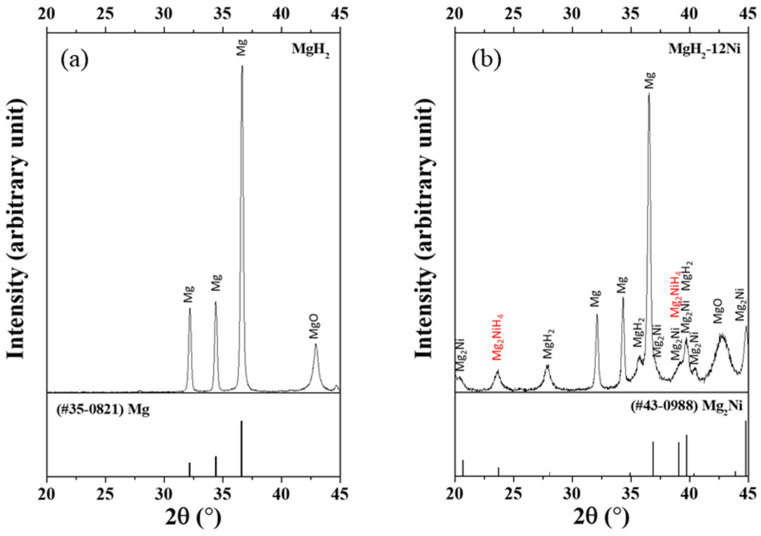
XRD patterns at room temperature of (**a**) milled MgH_2_ and (**b**) MgH_2_-12Ni after the reaction with CH_4_ at 12 bar and 773 K for 1 h and desorption under 1.0 bar at 773 K for 1 h.

Figure 1 shows the XRD patterns at room temperature of milled MgH_2_ and MgH_2_-12Ni after the reaction with CH_4_ at 12 bar and 773 K for 1 h and desorption under 1.0 bar at 773 K for 1 h.

When the milled MgH_2_ was heated to 773 K under 1.0 bar CH_4_ and vacuum pumped, the hydrogen in the milled MgH_2_ is thought to have been removed. It is believed that Mg_2_Ni was formed during heating to 773 K [22]. When the MgH_2_-12Ni was heated to 773 K under 1.0 bar CH_4_ and vacuum pumped, the hydrogen in the MgH_2_ is thought to have been removed.

The XRD pattern of milled MgH_2_ after the reaction with CH_4_ at 12 bar and 773 K and desorption under 1.0 bar at 773 K exhibited the Mg and MgO phases. The MgO is believed to have been formed during sample exposure to air to obtain the XRD pattern. This shows that the conversion of CH_4_ did not take place.

The XRD pattern of MgH_2_-12Ni after the reaction with CH_4_ at 12 bar and 773 K and desorption under 1.0 bar at 773 K exhibited the MgH_2_, Mg_2_NiH_4_, Mg, Mg_2_Ni and MgO phases. The formation of MgH_2_ and Mg_2_NiH_4_ indicates that the conversion of CH_4_ took place, the converted CH_4_ (hydrogen-containing mixture) is adsorbed on the particles, and MgH_2_ and Mg_2_NiH_4_ hydrides are thought to have been formed by the reaction of Mg (formed during heating to 773 K under 1.0 bar and vacuum pumping at 773 K) and Mg_2_Ni (formed during heating to 773 K) with hydrogen (formed via CH_4_ conversion and adsorbed on particles) during cooling to room temperature.

**Figure 2:**
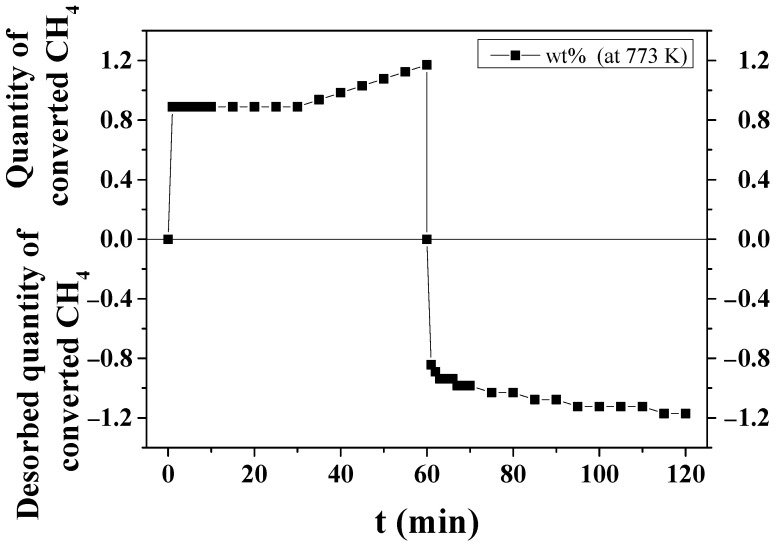
Quantity of converted CH_4_ versus time t under 12 bar CH_4_ at 773 K and desorbed quantity of converted CH_4_ versus t under 1.0 bar at 773 K for MgH_2_-12Ni.

Figure 2 shows the quantity of converted CH_4_ versus time t under 12 bar CH_4_ at 773 K and the desorbed quantity of converted CH_4_ versus t under 1.0 bar at 773 K for MgH_2_-12Ni. The quantity of converted CH_4_ under 12 bar CH_4_ at 773 K was 0.8 wt% for 1 min and 1.17 wt% for 60 min. The desorbed quantity of converted CH_4_ (hydrogen-containing mixture) under 1.0 bar at 773 K was 0.8 wt% for 1 min and 1.17 wt% for 60 min.

**Figure 3:**
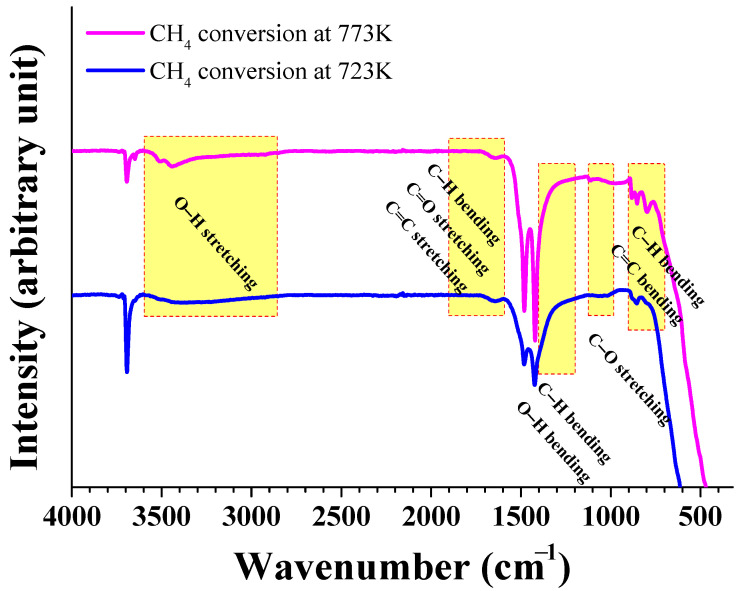
Attenuated total reflectance FT-IR spectroscopy (ATR-FTIR) spectra of MgH_2_-12Ni reacted with 12 bar CH_4_ at 723 K and 773 K, respectively.

Attenuated total reflectance FT-IR spectroscopy (ATR-FTIR) spectra of MgH_2_-12Ni reacted with 12 bar CH_4_ at 723 K and 773 K, respectively, are shown in Figure 3. Peaks for C-H bending, C=C stretching and C=C bending resulting from CH_4_ conversion were observed [23,24]. Peaks for O-H stretching, C=O stretching and C-O stretching are believed to be formed due to a reaction with oxygen in air.

**Figure 4:**
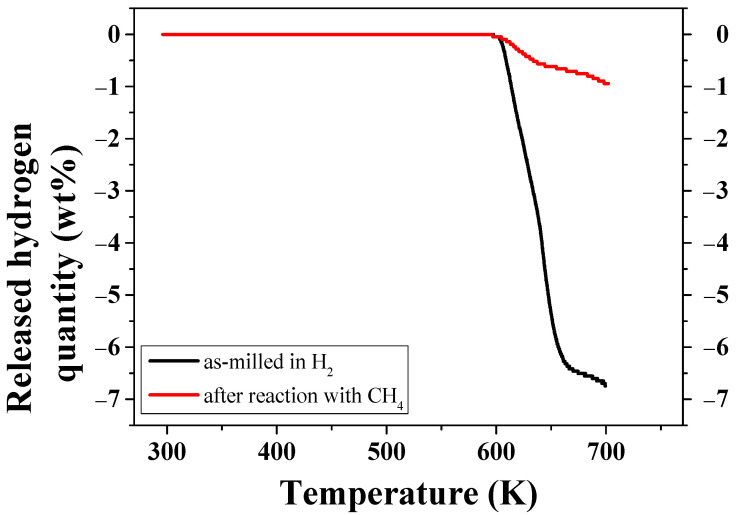
The curve of released hydrogen quantity as a function of temperature T for as-milled MgH_2_-12Ni and the curve of released gas amount as a function of T for MgH_2_-12Ni after the reaction with CH_4_ at 12 bar when heated at a heating rate of 5–6 K/min.

Figure 4 shows the curve of released hydrogen quantity versus temperature T for as-milled MgH_2_-12Ni and the curve of released gas quantity versus T for MgH_2_-12Ni after the reaction with CH_4_ at 12 bar when heated at a heating rate of 5–6 K/min. The as-milled MgH_2_-12Ni released hydrogen of 5.09 wt% up to about 648 K relatively rapidly, and slowly released hydrogen of 6.74 wt% up to about 700 K. MgH_2_-12Ni after the reaction with CH_4_ at 12 bar released gas (a hydrogen-containing mixture) of 0.66 wt% up to about 663 K rapidly and 0.94 wt% up to about 702 K slowly.

**Figure 5:**
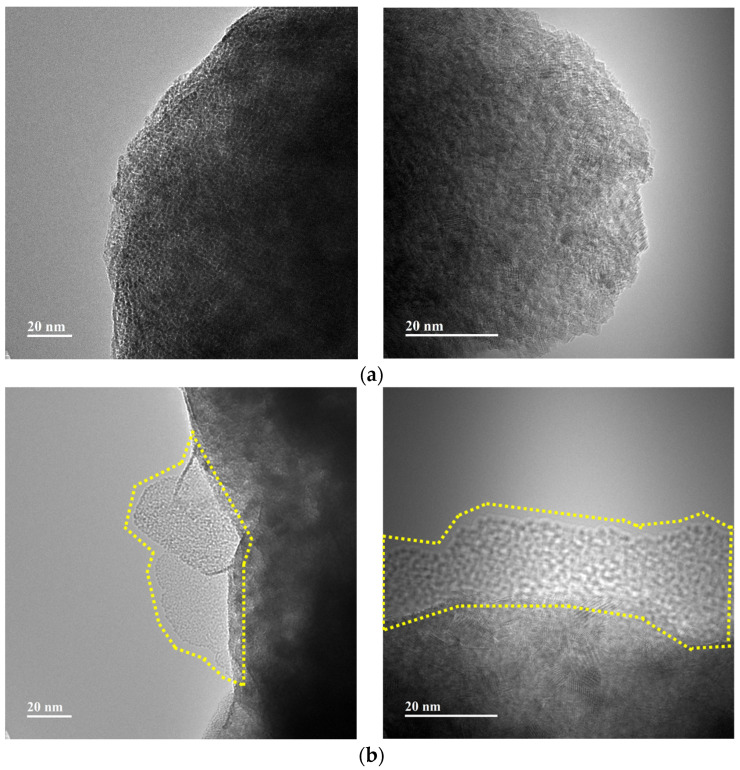
HR-TEM images of MgH_2_-12Ni (**a**) as-milled and (**b**) after reaction with CH_4_ at 12 bar and 773 K for 1 h.

Figure 5 shows HR-TEM images of MgH_2_-12Ni as-milled and after the reaction with CH_4_ at 12 bar at 773 K for 1 h. The as-milled MgH_2_-12Ni exhibits spherically shaped particles. The MgH_2_-12Ni after the reaction with CH_4_ shows carbon on the surface of the particles, which was highlighted.

**Figure 6:**
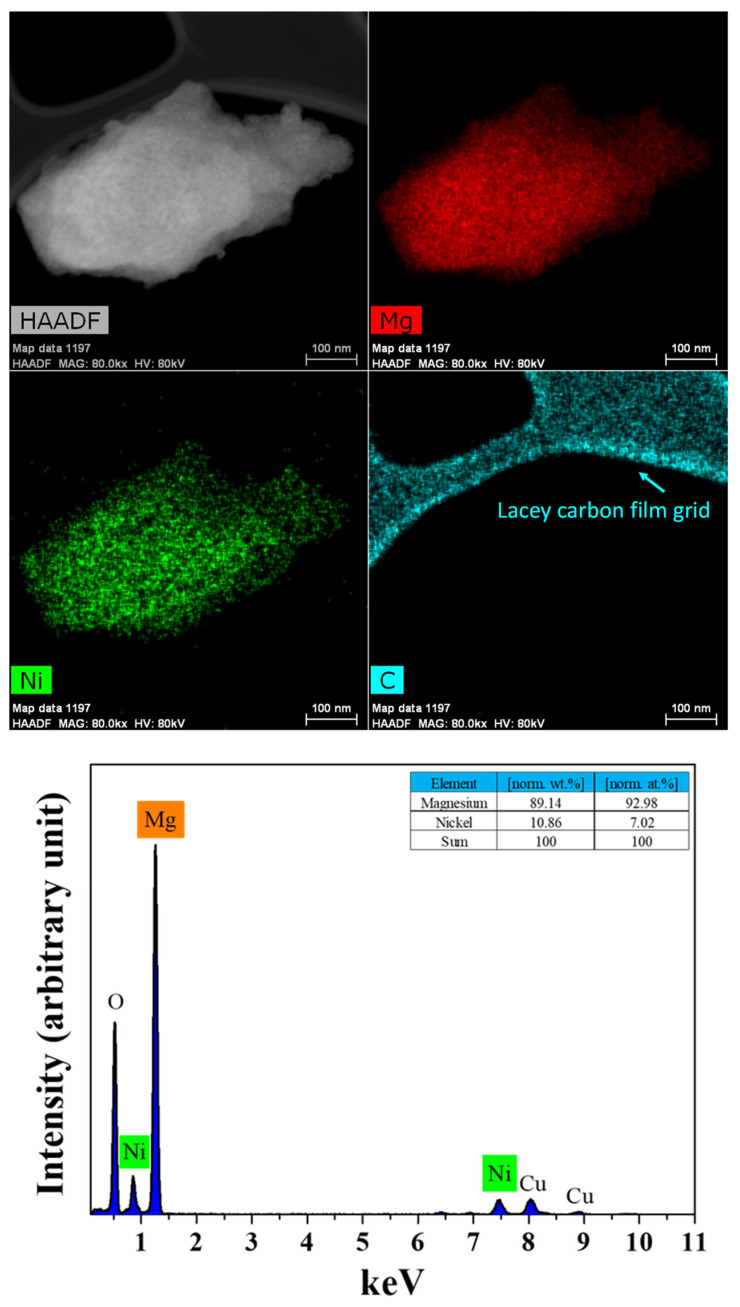
An HR-TEM image, EDS images and an EDS spectrum of the as-milled MgH_2_-12Ni.

An HR-TEM image, EDS images and an EDS spectrum of the as-milled MgH_2_-12Ni are shown in Figure 6. The EDS images show that the distribution of Mg, Ni and C on the particle is quite homogeneous. The oxygen is introduced due to exposure to ethanol and air. The particles were dried in air for 2 h after placing the sample particles on a Lacey carbon-supported copper grid, which were sonicated in an ethanol-filled vial. The EDS spectrum exhibits the peaks of Mg and Ni together with the peaks of Cu and O. The Cu peak appears due to the copper in the Lacey carbon-supported copper grid.

**Figure 7:**
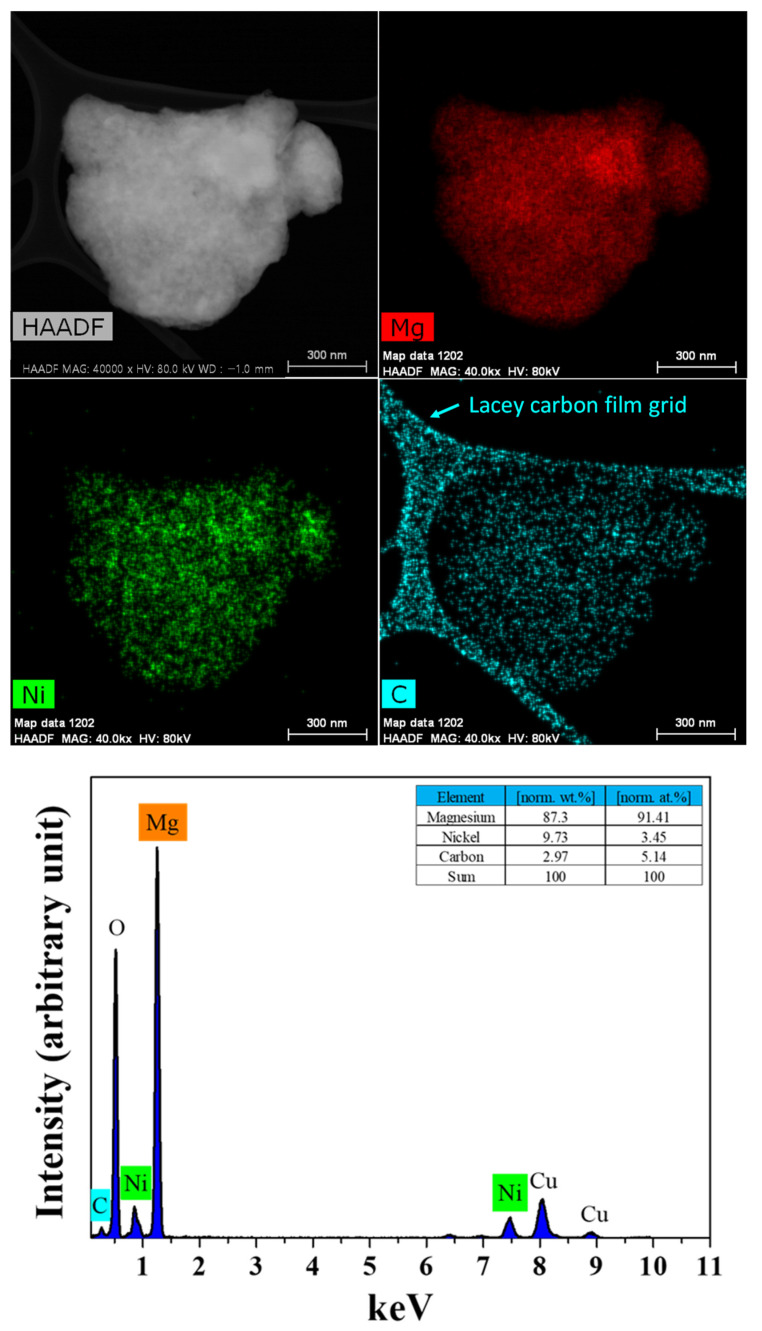
An HR-TEM image, EDS images and an EDS spectrum of the MgH_2_-12Ni after the 12 bar CH_4_ reaction at 773 K for 1 h.

Figure 7 shows a HR-TEM image, EDS images and an EDS spectrum of the MgH_2_-12Ni after the 12 bar CH_4_ reaction at 773 K for 1 h. The EDS images show that the distribution of Mg, Ni and C on the particle is quite homogeneous. The EDS spectrum exhibits the carbon peak together with the peaks of Mg, Ni, Cu and O. 

**Figure 8:**
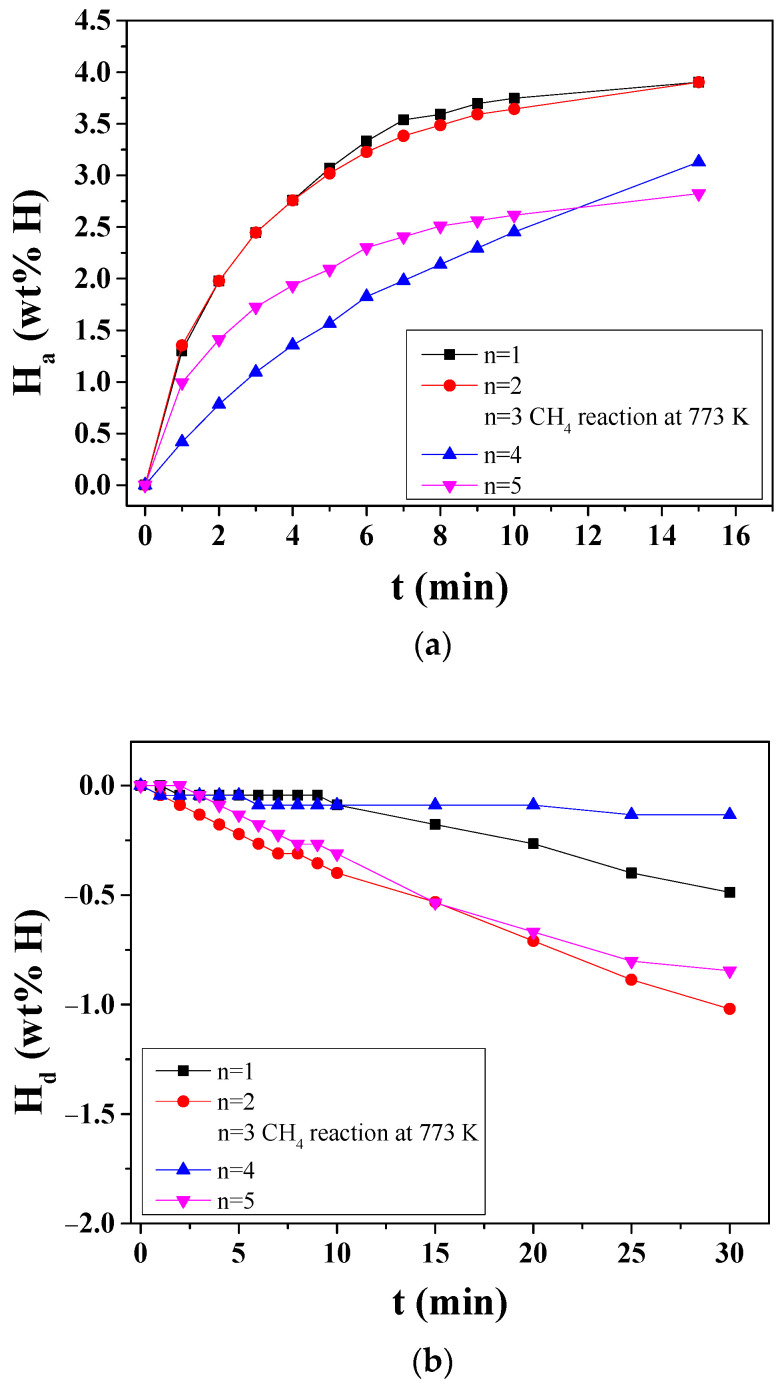
(**a**) Change in absorbed hydrogen quantity H_a_ versus time t curve under 12 bar H_2_ and (**b**) change in released hydrogen quantity H_d_ versus t curve under 1.0 bar H_2_ at 573 K with cycle number *n* for MgH_2_-12Ni. At *n* = 3, the MgH_2_-12Ni was reacted under 12 bar CH_4_ at 773 K and desorbed under 1.0 bar CH_4_ at 773 K.

The change in absorbed hydrogen quantity H_a_ versus time t curve under 12 bar H_2_ and the change in released hydrogen quantity H_d_ versus t curve under 1.0 bar H_2_ at 573 K with cycle number *n* for MgH_2_-12Ni are shown in Figure 8. At *n* = 3, the MgH_2_-12Ni was reacted under 12 bar CH_4_ at 773 K and desorbed under 1.0 bar CH_4_ at 773 K. From *n* = 1 to *n* = 2, the initial hydriding rate and the quantity of hydrogen absorbed for 60 min increased very slightly; the H_a_ versus time t curves at *n* = 1 and *n* = 2 were very similar. From *n* = 2 to *n* = 4, the initial hydriding rate and the quantity of hydrogen absorbed for 60 min decreased at lot. This means that the surfaces of MgH_2_-12Ni particles were contaminated with C and CH_4_; C and CH_4_ were adsorbed on the surfaces of MgH_2_-12Ni particles. From *n* = 4 to *n* = 5, the initial hydriding rate increased and the quantity of hydrogen absorbed for 60 min decreased a little, showing that the C and CH_4_ adsorbed on the surfaces of MgH_2_-12Ni particles were removed; the surfaces of MgH_2_-12Ni particles were recovered during pumping out after dehydriding. The decrease in the quantity of hydrogen absorbed for 60 min suggests that sintering of particles took place during hydriding–dehydriding cycling. From *n* = 1 to *n* = 2, the initial dehydriding rate and the quantity of hydrogen released for 30 min increased a lot; the incubation period for dehydriding, which appeared at *n* = 1, disappeared at *n* = 2. From *n* = 2 to *n* = 4, the initial dehydriding rate and the quantity of hydrogen absorbed for 30 min decreased a lot. This means that C and CH_4_ were adsorbed on the surfaces of MgH_2_-12Ni particles. From *n* = 4 to *n* = 5, the initial dehydriding rate increased a little (the incubation period for dehydriding was decreased from 9 min to 3 min) and the quantity of hydrogen absorbed for 30 min decreased a lot, showing that the C and CH_4_ adsorbed on the surfaces of MgH_2_-12Ni particles were removed. The results in Figure 8 show that the surfaces of MgH_2_-12Ni particles were contaminated with C and CH_4_; C and CH_4_ were adsorbed on the surfaces of MgH_2_-12Ni particles after the reaction with 12 bar CH_4_ at 773 K.

Figure 2 shows that the methane conversion proceeds quite rapidly (0.8 wt% for 1 min) and then proceeds very slowly to 1.17 wt% up to 60 min. The average particle sizes of milled MgH_2_ and MgH_2_-12Ni, which were measured via particle size analysis, were 1.39 and 0.65 µm, respectively. From these values, the specific surface areas of milled MgH_2_ and MgH_2_-12Ni were calculated to be 2.98 and 5.73 m^2^/g, respectively, assuming that the particles were spherical. MgH_2_-12Ni has a fairly large specific surface area (1.9 times), compared with milled MgH_2_. The distribution of Ni, which was observed by means of EDS (Figure 6 and Figure 7), was quite homogeneous.

The surface of MgH_2_-12Ni is very reactive because it was prepared by means of milling in a hydrogen atmosphere and heating in hydrogen to 773 K. Thus, CH_4_ was converted very rapidly at first. However, the CH_4_ conversion was very slow, at 1.17% after 60 min, and the conversion rate was quite low. We think that the conversion rate and the converted quantity should be increased. In future research, the composition of the MgH_2_-12Ni will be varied, the milling conditions will be changed and different CH_4_ pressures will be exerted. In addition, the variability of CH_4_ conversion depending on the number of cycles will be studied.

The pressure–composition isotherms (P-C-T diagram) in metal–hydrogen systems exhibit equilibrium plateau pressures at various temperatures. The equilibrium plateau pressures are the equilibrium hydrogen pressures at which the metal and hydrogen coexist in equilibrium. In order to form a metal hydride at a certain temperature, hydrogen with a pressure higher than the equilibrium plateau pressure must be applied. At a temperature of 773 K, the equilibrium plateau pressures of the Mg-H system and the Mg_2_Ni-H system are much higher than 12 bar, which was applied in the present work. The equilibrium plateau pressure at 773 K is 136 bar for the Mg-H system [25] and 98 bar for the Mg_2_Ni-H system [26]. It is therefore considered that Mg and Mg_2_Ni hydrides are not formed upon reaction with CH_4_ at 12 bar and 773 K. CH_4_ is converted and the converted gas mixture is adsorbed on MgH_2_-12Ni particles, and Mg and Mg_2_Ni hydrides are formed during cooling to room temperature as a result of the reactions of Mg and Mg_2_Ni with adsorbed hydrogen. The equilibrium plateau pressure is 1 bar at 557 K for the Mg-H system [25] and at 527 K for the Mg_2_Ni-H system [26]. At temperatures from 473 K to room temperature (during cooling), the equilibrium plateau pressures of the Mg-H and Mg_2_Ni-H systems are very low and the formation of MgH_2_ and Mg_2_NiH_4_ is possible.

The XRD pattern of milled MgH_2_ after the reaction with CH_4_ at 12 bar and 773 K and desorption under 1.0 bar at 773 K exhibited no MgH_2_ and Mg_2_NiH_4_ phases. However, the XRD pattern of MgH_2_-12Ni after the reaction with CH_4_ at 12 bar and 773 K and desorption under 1.0 bar at 773 K exhibited MgH_2_ and Mg_2_NiH_4_ phases. This shows that CH_4_ conversion took place, the converted CH_4_ (a hydrogen-containing mixture) was adsorbed onto the particles, and MgH_2_ and Mg_2_NiH_4_ hydrides were believed to be formed by the reaction of Mg (formed during heating up to 773 K under 1.0 bar and vacuum pumping at 773 K) and Mg_2_Ni (formed during heating up to 773 K) with hydrogen (formed as a result of CH_4_ conversion and adsorbed on the particles) during cooling to room temperature.

Ni was not observed in the XRD pattern of MgH_2_-12Ni after the reaction with CH_4_ at 12 bar and 773 K and desorption under 1.0 bar at 773 K. It is known that a small amount in the sample is not observed in the XRD pattern. We believe that Ni is present in MgH_2_-12Ni after heating to 773 K.

The addition of Ni for sample preparation is thought to lead to different results for the particles. The surface state of MgH_2_-12Ni and the greater surface area of MgH_2_-12Ni than milled MgH_2_ might have played a role in converting CH_4_. However, Ni and Mg_2_Ni formed during heating to 773 K are believed to have produced catalytic effects in CH_4_ conversion, playing a larger role in CH_4_ conversion. It has been reported that most methane-reforming methods usually use nickel as a catalyst [20].

Transition metals such as Ni are reported to have a catalytic effect on gas adsorption [27]. The addition of Ni (and less possibly Mg_2_Ni) could help CH_4_ to adsorb onto the particles.

The process developed in the present work is one in which the conversion of CH_4_, the storage of hydrogen and the separation of the remaining CH_4_ (by pumping out at room temperature) are all achieved in a single process.

In our future research, gas chromatography analysis will be performed on gases obtained after a reaction with CH_4_ at 12 bar and 773 K. This will help to verify the present work.

## 4. Conclusions

The conversion of CH_4_ to hydrogen and hydrogen storage was studied using a magnesium-based alloy. MgH_2_-12Ni (with the composition of 88 wt% MgH_2_ + 12 wt% Ni) was prepared in a planetary ball mill under high-purity hydrogen gas. The XRD pattern of MgH_2_-12Ni after reaction with CH_4_ at 12 bar and 773 K and desorption under 1.0 bar at 773 K exhibited MgH_2_ and Mg_2_NiH_4_ phases. This shows that conversion of CH_4_ occurred, the converted CH_4_ (hydrogen-containing mixture) was then adsorbed on the particles, and hydrides were formed during cooling to room temperature. The Ni and Mg_2_Ni formed during heating up to 773 K are believed to have brought about catalytic effects for converting CH_4_. MgH_2_-12Ni adsorbed 0.8 wt% of converted CH_4_ within 1 min in a reaction with CH_4_ at 12 bar and 773 K and then desorbed 0.8 wt% of converted CH_4_ within 1 min under 1.0 bar and 773 K. Attenuated total reflectance FT-IR spectroscopy (ATR-FTIR) spectra of MgH_2_-12Ni after reactions under 12 bar CH_4_ at 723 K and 773 K showed peaks of C-H bending, C=C stretching, O-H stretching, O-H bending and C-O stretching. In our future research, gas chromatography analyses will be performed on gases obtained after reactions with CH_4_ at 12 bar and 773 K. This will help to verify the present work.

## Data Availability

The data presented in this study are available on request from the corresponding author.
